# *Enterococcus gallinarum* as a component of the Autoinfectome: the gut-liver-autoimmune rheumatic disease axis is alive and kicking

**DOI:** 10.31138/mjr.29.4.187

**Published:** 2018-12-18

**Authors:** Dimitrios P. Bogdanos, Lazaros I. Sakkas

**Affiliations:** Department of Rheumatology and Clinical Immunology, University General Hospital of Larissa, Faculty of Medicine, School of Health Sciences, University of Thessaly, Greece

**Keywords:** autoimmunity, autoimmune hepatitis, infection, microbiome, microbiota, systemic lupus erythematosus

In recent years, we have repeatedly underlined the necessity to conduct meticulous assessments of the hierarchy of events that lead to the induction of autoimmunity through the close and complex interaction of the microbiota with a host who is prone to develop autoimmunity.^1–3^ We recently introduced the concept of infectome and autoinfectome to describe those infections throughout life that cause autoimmune diseases in genetically susceptible individuals, who have impaired immunoregulatory mechanisms.^4–7^ The events that take place before the establishment of infectious-related autoimmune disease largely rest on the microbiome changes of an individual over time. We proposed that environmental factors, which alter the normal microbiota may promote the growth of pathobionts which facilitate the development of autoreactive responses; such microbiota alterations may also impact on the ability of the host to suppress autoimmunity due to their direct or indirect effect on the functional regulatory T and B lymphocyte populations.^7^ In a recent paper by Manfredo Vieira et al.^8^ published in Science, *Enterococcus gallinarum*, a Gram-positive gut pathobiont could translocate, under a state of a gut barrier breakdown, into a systemic organ (liver) and subsequently induce experimental autoimmune disease in genetically susceptible mice; namely, systemic lupus erythematosus. The translocating bacterium induced skewed T helper cell differentiation, and colonized the liver. These two main effects led to neoantigens and pro-inflammatory cytokines induction which promoted the production of autoantibodies and the development of autoimmune disease.

*E. gallinarum* had the potential for translocation in (NZW × BXSB) F1 mice but not in C57BL/6 mice under pathogen-free conditions. Thus, the authors tested translocation in germ-free C57BL/6 mice monocolonised with *E. gallinarum.*^8^ Their data were intriguing. When competing microbiota were absent, *E. gallinarum* was able to induce a) intestinal barrier leakage; b) de novo production of ANA (anti-dsDNA) autoantibodies, and c) translocation to mesenteric veins, mesenteric lymph nodes, and livers of non-autoimmune C57BL/6 mice. Of interest, no autoantibody production was seen in C57BL/6 mice during monocolonisation with other bacteria that either remained in the gut (*B. thetaiotaomicron*) or translocated to tissues (*E. faecalis*). In search of the immunological mechanism which perpetuate autoimmune phenomena, the researchers found that colonization with *E. gallinarum* alone induced Th17 cells in the small intestinal lamina propria and mesenteric lymph nodes of C57BL/6 mice. These pro-inflammatory cells disappeared after vancomycin treatment. When this pathobiont was selectively depleted, through intramuscular vaccination using heat-killed *E. gallinarum*, mice exhibited reduced levels of serum autoantibodies and had prolonged survival. Such an effect was specific for *E. gallinarum*, as vaccination against *E. faecalis* or *B. thetaiotaomicron* did not influence autoantibodies or disease activity.^8^ Vaccination against *E. gallinarum* also prevented translocation. This was clearly documented by the lack of growth of *E. gallinarum* in internal organs.^8^

The fact that antibiotic treatment of bacterium-specific vaccination can cease autoimmune phenomena is a direct proof of the pivotal role of this specific microbe in the induction of autoimmune disease. As for all other infectious agents that have been directly linked to experimental autoimmune diseases, antibiotic treatment rather than immune suppression appears to be the proper treatment. These results were obtained in an experimental model of autoimmune disease. Arguably, the question arises if such data have any direct implication for the human disease.

SLE patients have evidence of a ‘leaky gut’; i.e. an impairment of gut barrier. When the authors assessed whether these patients have evidence of *E. gallinarum* translocation to human livers in patients with SLE (and concurrent autoimmune hepatitis [AIH]), 3 out of 6 SLE patients were positive for *E. gallinarum*, while none of the 6 controls obtained from healthy liver transplant donors with normal liver histology had evidence of the bacterium. These findings further strengthen the importance of this bacterium in the induction of the disease, SLE and/or autoimmune hepatitis. The co-occurrence of autoimmune hepatitis and SLE is not a frequent phenomenon. However, several patients with autoimmune hepatitis have anti-dsDNA antibodies. It would be of interest to check those patients’ livers for bacterium translocation. Most of the patients with newly diagnosed AIH have liver biopsies done at presentation for diagnostic purposes, and investigation in this biological material is feasible and rather easy to perform. AIH affects children, as well as adults, and extension of such an investigation to children may shed light on the hierarchy of the complex host-microbiota interactions paying attention to *E. gallinarum*.

A more relevant question would be if we need to search for evidence of *E. gallinarum* in all newly diagnosed patients with SLE, and if positive patients need treatment with antibiotics. In an animal model of a lupus-prone mice, vancomycin, initiated at 9 weeks of age, could attenuate lupus-like symptoms, reduce levels of IgG anti-dsDNA antibodies, diminish proteinuria, and decrease renal histopathological score. Such an effect was not seen with neomycin, an antibiotic with a broad spectrum of activity against both Gram-positive and Gram-negative bacteria. Vancomycin does not appear to trigger lupus-like disease in humans. However, vancomycin has been considered a triggering factor of linear IgA bullous dermatosis, a rare autoimmune blistering disease and its exacerbating role in autoimmunity cannot be neglected. Though *Enterococcus gallinarum* is present in the normal flora in human and animal guts, the growing usage of broad-spectrum antibiotics increased the prevalence of infections caused by *E. gallinarum*, gradually leading to multi-drug resistance and nosocomial infections mainly affecting the urinary tract, abdominal and biliary tract;^11^ its ability to cause human autoimmunity remains to be seen. In conclusion, the recent findings of Manfredo Vieira et al.^8^ are rather provocative and need meticulous assessment in relevance to their implication in human disease. If *E. gallinarum*, a gut bacterium, is proved to be an important trigger of extra-intestinal autoimmune disease, we will need to reconsider the hypothesis of infectious-driven autoimmunity in the context of the autoinfectome.^6,12^
